# Decision makers’ perceptions on implementing welfare technology in Swedish municipal elder care: a qualitative study

**DOI:** 10.1186/s12877-025-06102-5

**Published:** 2025-06-09

**Authors:** Johannes Österholm, Vedrana Baric, Ingrid Hellström, Katarina Baudin, Åsa Larsson Ranada

**Affiliations:** 1 Department of Health, Medicine and Caring Sciences, Division of Prevention, Rehabilitation and Community Medicine (PRNV), Unit of occupational therapy, Linköping University, Linköping, Sweden; 2 Department of Health Care Sciences, Marie Cederschiöld University, Stockholm, Sweden; 3 Department of Neurobiology, Care Sciences and Society, Karolinska Institutet, Huddinge, Sweden

**Keywords:** Digital solutions, E-health, Gerontechnology, Older person, Self-help equipment

## Abstract

**Background:**

Welfare technology is often depicted as a potential solution for managing the increasing challenges on elder care services, which is expected to rise due to demographic changes. In this study, we explore local decision-makers’ perceptions on implementing welfare technologies in Swedish municipal elder care.

**Methods:**

Four politicians and twelve local government officials were interviewed, and reflexive thematic analysis was utilized.

**Results:**

Our findings suggest that welfare technology is portrayed as a sustainable long-term solution, governed by bureaucratic structures. Successful implementation requires shared responsibility among various stakeholders but also moving in-between resistance and acceptance among staff and older people. Furthermore, welfare technology was viewed to reduce staff burdens and increase independence and participation for older people.

**Conclusions:**

Welfare technology is viewed positively by local decision-makers as a long-term solution to challenges associated with funding and staff shortages in elder care. It is expected to reduce the burden on staff and increase independence and participation for older people. However, bureaucratic challenges must be addressed to enhance the quality of elder care.

## Introduction

In Sweden and worldwide, a demographic shift is occurring [1, 2] challenging the organization of elder care. The key issues include a growing population of older people with complex care needs, technological advancements enabling prolonged care, and a decreasing working-age population to support and fund the welfare system [1, 3, 4]. Welfare technology is often presented as part of the solution to handling these challenges, both in terms of providing elder care services and ensuring efficient use of available resources. With the development of welfare technology, smart technologies and artificial intelligence, people’s use of digital technologies will continue to create new practical solutions for elder care. New forms of organization and communication for public, private, and social activities will characterize all sectors of society, not least health and elder care [5, 6].

Welfare technology is a concept primarily used in a Nordic context [7] that broadly refers to various technologies and devices used in different care contexts such as medicine dispensers, GPS-trackers and digital calendars. Welfare technology can be given as assistance, prescribed as an aid, or purchased on the consumer market, affecting which technologies that are considered as welfare technology internationally [8]. Thereby, there is no official international definition of welfare technology. The point of departure of this study is the definition of Welfare technology provided by the Nordic Welfare Center as “all technology which in one way or another improves the lives of those who need it. Technology is used to maintain or increase security, activity, participation or independence for people with a disability or the elderly” [9]. Welfare technology can be used by the person themselves, by significant others or by staff.

Previous research highlights various benefits of using welfare technology for older people such as facilitating aging in place [10, 11], support with self-care [12], monitoring symptoms [13] and increased independence [14–16]. Barriers to usability have been identified, considering procurement practices [13, 17], costs, privacy, and security reasons [10]. Research has also shown that for welfare technology to be used in elder care there must be an actual need for the technology [15, 18]. The importance of a social network to facilitate and support the usage of welfare technology amongst older people has also been highlighted [10, 19].

Studies in this area have mainly focused on the implementation of specific welfare technology [14, 20]. The primary goal of previous research has been to assess the effects of various products, rather than to understand the benefits for users or to address the needs of end-users [21–23]. More knowledge is needed about how the use of welfare technology– as a broader phenomenon encompassing various technologies used in elder care– is influenced by the expectations and beliefs of different stakeholders’ such as older people, significant others [10, 14], staff working in elder care [11], and from an organizational perspective with decision-makers and/or policymakers [24, 25].

Earlier studies on policymaking portray a discrepancy that exists between decision-makers, frontline staff, and older people [14]. Decision-makers seem to be more optimistic about the implementation of welfare technologies in elder care than other stakeholders [14, 18, 26]. This seems to be related to a more positive discourse of how to solve various challenges in the organization of elder care, in which critical issues regarding welfare technology are often marginalized [18, 26, 27]. Further, policies and regulations on welfare technology span between different sectors and departments and involve many stakeholders both internally and externally to the organization, challenging collaborative efforts [24]. The successful integration of welfare technology in the everyday life of and care for the older population depends significantly on policies and services that ensure equitable access and use, but research that supports policymakers to enhance implementation is lacking [24, 25].

In summary, earlier research [24, 25] has shown that policymakers’ understanding and incentives of welfare technology depends on both their willingness and investments. These efforts are facilitated by collaborative and cooperative actions that span between different sectors and departments. As significant resources are invested in welfare technology and there is considerable optimism surrounding its implementation, embedded in complex organizational structures [18, 26], there is a need to better understand welfare technology as a broader phenomenon in elder care from the perspective of decision-makers (i.e., local politicians and local government officials). Therefore, this study aims to explore decision-makers' perceptions on implementing welfare technologies in Swedish municipal elder care.

## Methodology and data

This study utilizes a qualitative research design to explore the complexity of local decision-makers’ perceptions on implementing welfare technologies in municipal elder care. Embedded in the research tradition of constructivism, this approach explores participants’ subjective experiences and explicit and implicit perceptions within their societal contexts, based on thematic analysis [28–30].

This study has been designed and conducted in accordance with the Declaration of Helsinki [31] and is reported according to Standards for Reporting Qualitative Research (SRQR) guidelines [32]. The researchers involved in this study were four females and one male. The participating researchers´ have research backgrounds in various areas such as welfare technology, municipal elder care, and clinical research involving older people, significant others, staff, and decision-makers.

### Recruitment

Participants were recruited using a strategic sampling procedure [33] based on the following criteria: local politicians or local government officials with the responsibility of making strategic decisions regarding the implementation of welfare technology in municipal elder care, and more than one year of experience in their current position. They were recruited from various municipalities with different population sizes and at various strategic levels to get diversified data material. Data collection occurred between January and November 2024.

In the recruitment process of participants, written information was provided through e-mail about the aim of the present study and the study procedure. All interviews were conducted through a video call. All participants were given oral information about the study before the interview began. Informed consent was given in writing through mail or orally documented through a separate audio recording.

### Participants and study context

In total, 16 participants decided to participate in the present study. The working tasks of the officials were on different strategic levels (implementation of welfare technologies between municipalities at the regional level, IT-security, coordinator and development leader of welfare technology, and responsibility to educate and support staff in utilizing various technologies) (Table 1 participant information). Currently, none of the participants were working with providing elder care services directly to an older person, but some had previous experience of working as an assistant nurse, or as an occupational therapist.

**Table 1:** Background information of the participants, *n* = 16

Sex, n:	
Women	12
Men	4
Local government officials, n:	
Politicians	4
Local government officials	12
Highest level of education, n:	
University	10
Upper secondary school	6
Area of Higher Education	
Health Care	2
Social Care	4
Information technology	3
Other	1
Working experience in current position, n:	
<5 years experience	8
>5 years experience	8
Size of municipalities, n:	
Small rural municipality (< 10 000–45 000 inhabitants)	5
Larg municipality (> 100 000-170 000 inhabitants)	11

The responsibility for elder care in Sweden is divided between municipalities and regions, governed by local politicians with considerable autonomy, and administered by local government officials [34, 35]. Due to the complexity of the Swedish elder care system, these authorities need to coordinate closely to provide integrated care [36]. Currently, Sweden is transitioning to integrated care, aiming to establish person-centered, cohesive, proactive, and health-promoting services [37]. Welfare technology is central to this transition, enhancing independence, activity, safety, and participation among older people. This division of responsibility and transition to integrated care may affect decision-makers’ perceptions of implementing welfare technologies in elder care.

Five participants from smaller rural municipalities mentioned that implementing welfare technology was a minor part of their job and they had few or no colleagues working with the implementation of welfare technology. In contrast, officials from larger municipalities reported working closely with colleagues, often focusing solely on welfare technology implementation.

### Data collection

Individual semi-structured interviews [38] were conducted. All interviews were audio recorded with an external dictaphone to ensure that data was stored appropriately on secure servers. After each interview, the recordings were transcribed verbatim by the interviewer. Besides questions about demographic information about the participants, questions in the semi-structured interview guide revolved around implementation of welfare technology and their role as decision-makers in this process, overall purpose of implementing welfare technology in elder care, and what they perceived to be the prevailing ideas about welfare technology in elder care. The same interview guide was used for all participants and can be accessed through the study protocol [39]. The duration of each interview was on average 50 min (range 34–71 min).

### Data analysis

Reflexive thematic analysis [28–30, 40] served as the analytical framework for this study. There are some analytical phases that are central to reflexive thematic analysis that guided the process: familiarization with data, data reduction (i.e., extracting sequences from the data of relevance for the aim of the study), condensation, semantic coding, and constructing a thematic representation of the phenomenon of interest. The first, second, and last authors read the transcripts to become familiarized with the data and discussed these to get a comprehensive understanding of their contents.

In the continued analysis, Nvivo^®^ was utilized. Sequences of relevance were extracted from the data by the first author. These sequences were then condensed and coded (see Table 2). This process was conducted inductively based on what the participants conveyed in the interviews (i.e., semantic coding). To ensure trustworthiness, these preliminary themes were then developed further through continued analysis and discussions between JÖ and VB. Some themes were combined and reorganized based on shared meanings. Dependability was achieved through reflexive practices during data collection and analysis, enhanced by an audit trail of decisions and initial thoughts on codes and themes. All preliminary themes were checked against the coded extracts and the entire dataset (VB, ÅLR). The final themes were refined through joint discussion in the research group, generating descriptions and summaries for each theme. In the results section, six themes are presented regarding local decision-makers’ perceptions on implementing welfare technologies in municipal elder care. Quotations from the interviews are utilized in the results section to exemplify the presented analysis and to make it possible for readers to make their own assessment of the results trustworthiness. These quotations have been pseudonymized and translated from Swedish into English by the authors.

**Table 2:** Example of data extract with applied codes and theme

Data extract	Coded for	Theme
*“I think many people are afraid of technology*,* that it will take over their jobs. Now this thing is going to do something that I have been doing all the time.” (Official*,* small municipality 2)*	*Suspicious of the technology* *Being replaced by technology*	***Moving in-between acceptance and resistance***
*“We also have many volunteer organizations connected to seniors meeting place. We are exploring the possibility of volunteers providing social companionship through digital solutions for those who cannot come to a senior meeting place” (Government official*,* small municipality 5)*	*Increased collaboration with end-users* *Active engagement and collaboration with the community*	***Establishing a sharedresponsibility for utilizing welfare technology***

## Results

In the results section, we present six themes that were pivotal for the implementation of welfare technology, as highlighted by local decision-makers in municipal elder care. Welfare technology was seen as a necessary means for sustainable and long-term solutions (1), governed by various bureaucratic structures (2). Successful implementation requires establishing shared responsibility among various stakeholders (3) and managing the movement between resistance and acceptance among staff and older people (4). Welfare technology has the potential to reduce work-related burdens on staff (5) while simultaneously increasing independence and participation in everyday activities for older people (6). Themes and key findings are presented in Table 3.

**Table 3:** Themes and key findings

Themes	Key findings
Welfare technology as a necessary mean for sustainable and long-term solutions	- Welfare technology can improve operational efficiency and organizational structure, staffing and economic challenges while enhancing service quality for end-users.
The encounter between welfare technology and bureaucratical structures	- Effective integration requires long-term strategies, financial support, and a comprehensive approach from procurement to usage. - Municipal bureaucracy can cause delays in implementation. - European and national regulations affect the effectiveness of implementation.
Establishing a shared responsibility for utilizing welfare technology	- Developing collaboration among municipal and regional healthcare services, providers, developers, and end-users is necessary. - Clearer collaboration and defined responsibilities are essential, with staff and older people working together to implement and use technologies. - Effective implementation requires community engagement.
Moving in-between acceptance and resistance	- Successful implementation requires acceptance from both organizations and end-users and providing staff with education and time to adapt. - Need to convince older people that technological solutions are suitable.
Welfare technology to reduce the burden on staff	- Welfare technology should complement, not replace, qualified staff, focusing on enhancing care quality rather than cost savings. - Technology can reduce staff time on administration.
Aspiration for increased independence and participation in everyday activities of older people	- Technology can empower older people to participate and manage everyday activities, safeguard privacy and, increase independence.

### Welfare technology as a necessary mean for sustainable and long-term solutions

Demographic changes challenge the organization and provision of elder care services, leading municipalities to seek strategic solutions for sustainable care. Welfare technologies are seen as essential for improving operational efficiency and organizational structures, addressing staffing and economic challenges. Developing a sustainable and long-term solution was deemed to become even more crucial in the future, with a growing older population with more extensive health and care needs to be addressed in municipal elder care:

*“To meet the healthcare challenges*,* welfare technology is needed. That is the main driving force*,* really. We must use technology*,* otherwise*,* there simply won’t be enough staff in the future.” (Official*,* large municipality 1)*.

In relation to this, participants, mostly in larger municipalities, stressed that welfare technology was one possible solution to address staffing issues and economic challenges, while enhancing the quality of services for end-users:

*“We saw early on in the administrative leadership that welfare technology can be a way for us to achieve operational development and organizational development. This not only solves the problem or challenge of staffing and economic issues but also adds quality for our users or customers. And this has led us*,* in this municipality*,* to build an approach to welfare technology where we link welfare technology with operational development. Fundamentally*,* that’s what it’s about.” (Politician*,* large municipality 1)*.

### The encounter between welfare technology and bureaucratic structures

To ensure sustainable solutions in municipal elder care, participants highlighted the need for long-term strategic implementation strategies, financial support, and encouragement from both political and administrative management. They emphasized the importance of an entire chain perspective, from procurement to usage, to ensure effective integration of welfare technology, leading to more efficient and effective care solutions. The participants emphasized the necessity to identify existing needs, whether for staff or older people, and match these needs with available technologies or developers who can create solutions. Participants stressed that technology should always aim to address real problems and meet existing needs within elder care to be truly effective and meaningful.

*“Instead of matching needs to the solutions already available on the open market*,* we say*,* ‘These are the needs of our end-users. What solutions are available for them?” (Official*,* small municipality 3)*.

Small municipalities were portrayed to often lack specialized project managers and resources for new welfare technologies. For example, controllers and IT support are too busy with daily tasks to prioritize projects about welfare technology, causing delays and challenges in managing and implementing welfare technologies. Participants stated that some projects like medication dispensers get stuck in municipal bureaucracy, causing delays in implementation due to the involvement of various stakeholders and the need for substantial investments in terms of economy and staff engagement. The focus on innovation procurement and staying at the forefront of innovation requires a huge commitment to continuously improving and updating the technologies used, to ensure that the best and most effective solutions are available. This commitment was described as greatly challenging for decision-makers, especially in smaller municipalities.

Stricter European and national regulations on digitalization were raised by a few participants as requiring municipalities to actively work on meeting requirements by ensuring the necessary digital infrastructure, while also continuing to progress in welfare technology implementation. Participants also argued that without national regulations and standardized implementation plans, staff may not receive uniform training and support, resulting in varying competencies and potentially impacting the effectiveness of implemented welfare technology:

*“We don’t really have*,* what should I say*,* a very clear implementation plan for welfare technology in general*,* but each project*,* if you say implementation project*,* that plans for the implementation and support*,* meaning what type of training that is available or how much support that is provided from the project manager or the project group*,* it largely depends on how big the effort needed is. So*,* there is no general*,* implementation strategy.” (Official*,* small municipality 4)*.

### Establishing a shared responsibility for utilizing welfare technology

To break current organizational boundaries in elder care, clearer collaboration and defined responsibilities between municipal and regional healthcare services are essential. Participants emphasized that rapid technological development and the current transition to integrated care in Sweden requires intense collaboration between stakeholders to ensure that welfare technologies are relevant and effective, meeting the needs of both staff and older persons. The implementation of welfare technology requires shared responsibility among municipal and regional healthcare services, developers, staff, and older people. This collective effort was seen as crucial for innovation and effectiveness. Therefore, long-term collaboration among stakeholders is essential for developing, testing, and implementing technologies in municipal elder care. Additionally, collaboration between different municipalities is necessary to develop infrastructure and use resources efficiently:

*“It is like when the boundary between what the municipality does and what the region does becomes increasingly unclear as we work together with the same patient/user*,* perhaps in teams composed of both municipal and regional staff. Where we sometimes use the region’s technology and sometimes the municipality’s technology with different areas of responsibility. And that is probably an area where I think we will have a lot of discussions in the future.” (Politician*,* large municipality 1)*.

Close collaboration with staff is important for practical insights into the needs and challenges faced by older people, ensuring that welfare technologies are safe, effective, and tailored to real-world elder care scenarios. This feedback is essential for strategic decisions regarding the implementation and procurement of technologies. Close collaboration with end-users, including significant others, who can provide valuable feedback based on their day-to-day experiences with technology was further needed.

*“Older persons*,* who are often the vulnerable group*,* their voices may not always be heard*,* or their perspectives may not always be seen. It’s important to visualize them. When I talk about different values being created*,* it’s beyond mere efficiency. It’s clear that I have realized that it is very important to include the needs and perspectives of seniors.” (Official*,* large municipality 6)*.

Effective implementation of welfare technology requires active community engagement. Understanding and addressing community concerns proactively can mitigate resistance against technology. Also, different communities may respond differently to welfare technology and therefore, tailoring the implementation plan to fit the specific needs and dynamics of each community can enhance the success. Feedback from older people was also described as important to support developers to refine and improve technologies. Participants emphasized collaboration with businesses and the private sector in the innovation procurement process, often at the prototype stage. There is a desire for developers to work closely with staff to customize and adapt technologies to meet specific user needs and regulatory requirements:

*“And then you have to look at whether there is a solution that would fit for this. And then*,* is it an interesting enough solution to test? And what kind of company is it? Are they interested in development and getting feedback on their solution?” (Official*,* large municipality 7)*.

### Moving in-between acceptance and resistance

Participants stressed that implementing welfare technology requires acceptance from organizations as well as end-users, including staff and older people, who may fluctuate between acceptance and resistance. Time, commitment, strategic planning, and ongoing support from all organizational levels were described as crucial to achieve maturity, where organizations are ready and capable of integrating welfare technologies into their operations.

*“We started introducing medical dispensers in 2017 as part of a project*,* we might have implemented them directly after the project ended. However*,* it often takes the first two to three years for the implementation to truly take hold*,* as it takes time for the organization to mature.” (Official*,* large municipality 9)*.

Participants with extensive experience noted that resistance to technology among staff has decreased over time. Most staff now appreciate technology if it functions as intended. Additionally, participants mentioned that staff understand that welfare technology aims to complement their work, not replace them, making tasks easier. Participants emphasized that for staff to accept welfare technology, education and time to adapt their working habits are essential. They noted that if technology is introduced too quickly, it won’t be used, and staff will revert to old procedures. Successful implementation requires developing the necessary conditions to ensure acceptance.

*“But we have had a preliminary study with a pilot for speech recognition*,* that is*,* to streamline record-keeping and documentation for our care managers […] this technology works quite well*,* and it helps me. But when it gets very stressful […] And that’s where I think people often fail*,* it’s not always a bed of roses to transition to new ways of working” (Official*,* small municipality 4)*.

Participants discussed the acceptance of welfare technology among older people. They emphasized that technology should not be imposed on older people. If someone has difficulty using technical solutions due to a lack of skills or disabilities, care staff should address their needs by offering complementary services. However, technical solutions should be encouraged as the first option if they can effectively meet the person’s care needs, reducing pressure on the elder care system. Participants described how they actively promoted technologies at senior forum, lowering tariffs for suitable technologies, and encouraging older people to buy technologies on the consumer market. These strategies aimed to convince older people that technological solutions were suitable and sometimes better than staff assistance. They also pointed out that older people need to be educated about various technologies to understand their benefits.

### Welfare technology to reduce the burden on staff

Welfare technologies were frequently described as a complement to qualified staff in elder care, improving operational efficiency, reducing the burden on professional staff, and enhancing the quality of care for older people. Most participants saw cost savings as a beneficial side effect, not the primary motivation for using welfare technology. Instead of reducing staff or cutting costs, the introduction of welfare technology was stressed as a way to use resources effectively to develop and strengthen elder care.

*“We haven’t had the requirement*,* which I think is an advantage*,* to constantly see the benefit in money from day one. Instead*,* we have had the opportunity to integrate the technology in such a way that we see it creates value for the individual*,* independence*,* and autonomy*,* but also value for our employees in the form of a better work environment*,* greater flexibility.” (Official*,* large municipality 10)*.

Participants highlighted technologies like automated medication dispensers and electronic health records that support routine tasks, reducing staff time on these activities. Digital keys and medication lists minimize travel between clients and the office, freeing up time for client care. Digital records keep staff updated on care plans, enhancing coordination and accurate information sharing.

*“In the short term*,* I don’t see technology replacing people*,* but technology can help*,* especially our employees*,* to be more efficient. It can serve as decision support or help quickly identify who needs assistance*,* thereby freeing up time that was previously spent checking on people who don’t need help at the moment. This means that the same number of people can spend more time on those who need care and less on those who don’t need it at the moment. So*,* the same number of people can provide more care*,* if I may put it a bit industrially*,* without having to work faster.” (Politician*,* large municipality 4)*.

### Aspiration for increased independence and participation in everyday activities of older people

As local decision-makers, participants aspired to implement welfare technology to facilitate continued participation in activities, empowering older people to manage everyday activities. Technology was described as safeguarding privacy and enhancing independence.

Encouraging the use of welfare technology can help older people maintain independence longer, positively affecting their health and reducing the burden on elder care systems. Medication dispensers were frequently mentioned as a means to ensure timely medication administration, supporting older people take their prescribed medication independently. This reduces their dependence on home care staff and alleviates anxiety about waiting for potentially delayed staff. Additionally, medication dispensers create a predictable daily structure, allowing older people to maintain their usual routines instead of adapting to the schedules of home care staff.

*“You don’t become as dependent on medication times*,* for example. The care recipient can manage their medication themselves*,* and if they have other visits*,* they can take it at any time during the day […] If they were told someone would come at 10 but they arrive at 10:30*,* they sit and wait*,* perhaps worried that no one will come at all. But with these […] medication dispensers*,* they can take their medication whenever they want*,* so they avoid that insecurity today.” (Official*,* large municipality 11)*.

Furthermore, technology was seen as a facilitator to safeguard privacy and increase independence in everyday activities. Automatic flushing and drying bidets were frequently mentioned for ensuring privacy in intimate situations, which, according to the participants, most older people wanted to manage themselves, thereby increasing their independence.

*“Then it is not always that it streamlines and saves a lot of time for the staff*,* but it may be completely different values for the person who benefits from the technology*,* so to speak. It leads to greater independence […] and then fewer services are needed.” (Official*,* large municipality 1)*.

## Discussion

The results of this study have shown that the main goals of implementing welfare technology in municipal elder care are to provide sustainable long-term solutions during times of financial concerns and to enhance the quality of care services. By optimizing staff allocation to ensure that staff are available where they are most needed, the overall efficiency and effectiveness of care can be improved. These opportunities can lead to better resource management and improved outcomes for both staff and older people.

Participants emphasized the need for a comprehensive approach, considering the entire chain from procurement to usage, to ensure that welfare technology is effectively integrated. This comprehensive approach creates opportunities for more efficient and effective care solutions, rather than treating these as separate elements. Furthermore, it is of importance that contracts for the procurement of welfare technology are formulated to allow flexibility in relation to technological developments. This ensures that the technology remains relevant and up-to-date, thus meeting the users’ needs and creating opportunities for continuous improvement.

Previous studies indicated that welfare technology often did not align with the needs or preferences of either the staff or older people [41]. Additionally, procurement procedures covering a technology rather than needs, and spanning several years, might lead to the technology becoming obsolete [18]. Our results suggest that various preconditions exist related to this in different municipalities, highlighting the challenges in this area. This shift in procurement of welfare technologies, as presented in the results of this study, might reflect the vision and national eHealth strategy [42] presented by government offices and the Swedish Association of Local Authorities and Regions [SALAR] [43], which seem to have a significant impact on how decision makers at various levels and in different municipalities form expectations on welfare technology in elder care. SALAR recently published a support-structure [44] on how to conduct an analysis of benefits when implementing welfare technology, covering various aspects: cost-benefit analysis, cost-benefit calculation, benefit realization, financial benefit, redistribution benefit, and quality benefit.

Our results acknowledged the importance of shared responsibility regarding the use of welfare technology among different stakeholders. This is also portrayed in the transition to integrated care that is ongoing in Sweden, which describes the use of welfare technology as a central part [37] where municipalities and regions must coordinate their services. It is also reflected in the two different legal frameworks (i.e., the Social Services Act (SoL) [45] and the Health Care Act (HSL) [46]) that govern elder care in Sweden. In practice, these laws can impact on the use of welfare technology as assistive technology in such a way that parts of the technical solution are prescribed, implemented, or used by regional personnel according to the HSL, while other parts are managed by municipal staff in accordance with the SoL. This is something that needs to be further explored and managed as the transition to integrated care continues.

In contrast to previous studies, which highlighted the potential intrusion of privacy by using various technologies such as sensors and night cameras collecting information about older people, this issue is not problematized in our results. When these technologies were implemented in elder care, much research revolved around ethical and integrity concerns [47]. However, aspects of intrusion are not at the fore front in our results. Instead, positive aspects of an older person’s privacy and independence in everyday situations are presented, such as managing toileting or taking medication by oneself, supported by various welfare technologies. This might be a consequence of the aim of this study, focusing on welfare technology as a phenomenon and not a specific technology where privacy issues might be more obvious. All participants in this study expressed positive views regarding welfare technology, highlighting its benefits. The inclusion of welfare technology in elder care was seen as a given, with no one questioning its necessity. However, participants raised questions about challenges related to the implementation of specific technologies in elder care. Whether this is dependent on technology per se or welfare technology as a phenomenon needs further investigation.

The results in our study suggest that there is a movement between acceptance and resistance in relation to welfare technology, presenting both opportunities and challenges that decision-makers are aware of and actively address to promote the use of technology among older people as well as staff. Hence, this study adds to existing knowledge the perspective of decisionmakers, who, according to our findings, acknowledge welfare technology as an important part of handling demographic challenges for the Swedish elder care system. Previous studies have presented a tendency of discrepancy between how users of technology (i.e., older people and staff) and decision-makers perceive the implementation of various technologies, where decision-makers seem to have a more positive stance [14].

Our findings suggest that decision-makers acknowledge the need for staff in elder care and the importance of facilitating their work by reducing routine or administrative duties so that personnel resources can be used where they are most needed. Previous studies partly support our findings, that various technologies could provide better working conditions for staff [5, 6], but also increase independence among older users [48]. In a previous study, smart sensors were argued to increase independence but may reduce social interaction [48]. Our findings suggest that decision-makers argue that technology should not replace human contact but rather be used strategically to free time for staff, time that could be used to ensure human contact when needed. Still, it is reasonable to assume that there is a risk that technology reduces actual human contact in the form of a physical meeting and might be replaced by various digital solutions. This is something that will be studied further in forthcoming studies [39].

Welfare technology and digitalization are intended to address the challenges closely connected to demographical changes that the Swedish elder care system must handle. Here, work tasks are supposed to be administered or supervised by technical solutions. The way health care personnel describe the challenges they face in implementing new welfare technology are similar across previous research, demonstrating that this is an international phenomenon [49–52]. Consequently, older people must therefore take responsibility for purchasing, updating, and handling various technological devices and solutions. This has been problematized previously in (Swedish) research arguing that not all older people are interested in using technology [15].

Our results are analyzed from the perspective of decision-makers and from their perspective, there seems to be various strategies to increase acceptance towards welfare technologies to catering to different needs. Anyhow, our results show that from a strategic perspective, those who are unable to use welfare technology should be offered more traditional services. Still, there is evidence from previous studies that significant others are an important stakeholder when technological solutions or devices are used in elder care [14, 53]. Consequently, there is a possibility that the formal responsibility of providing elder care services will be shifted to significant others. That is supported by previous research, which highlights the discrepancy in how the implementation of welfare technology is perceived more positively by decision-makers compared to other groups [14]. As there are no formal obligations in Sweden for significant others to provide elder care services, such as to one’s parents or spouses, there is a risk that the responsibility will be shifted unnoticed if they must support their loved ones with the use of various technologies. This presents both opportunities and challenges, as it could lead to increased involvement of family members in elder care, but also place additional burdens on them.

### Methodological consideration

In this study, decisionmakers at various levels in both larger and smaller municipalities participated. The intention of using a strategic recruitment process was to cover various perceptions on implementing welfare technology. Consequently, the participants’ experience of making decisions regarding the implementation of welfare technology ranges from strategic decisions to practical decisions in the actual implementation. Therefore, the data consists of political visions, as well as practical, day-to-day examples of participants’ experiences with welfare technology. In the analytical procedure, this discrepancy was used to create a more nuanced understanding of the phenomenon in question than would likely have been the case if the data had been analyzed separately based on politicians and local government officials. Furthermore, a strategical sampling procedure was utilized to recruit participants from various municipalities with different conditions in relation to inhabitants but also in relation to have several municipalities represented in various regions in Sweden to get a diversified material not dependent on a specific local context or way of organizing municipal elder care.

In the interviews, the participants raised several different perspectives from various stakeholders such as staff, older people, and the municipal organization. Nonetheless, few took the perspective of significant others in relation to the implementation of welfare technology. In previous research on various technologies in elder care, significant others have been portrayed as central stakeholders [10]. The lack of significant others in our data, as well as in our results, may be a consequence of not posing any direct questions regarding them. It might also be explained by the Swedish legislation governing municipal elder care, where the elder care user is the formal client who has the right to make decisions about services on their own. In future research, the perspective of significant others will be studied further [39].

Furthermore, the interviews were conducted by four authors (JÖ, VB, KB, ÅLR) following the same semi-structured interview guide. This has resulted in diversified data covering local decision-makers’ perceptions on the implementation of welfare technology in elder care, as the researchers have various research backgrounds and thereby steered the interview in various directions. In semi-structured interviews, there is an interaction between the interviewee and the interviewer that affects the direction the interview takes.

In relation to confirmability of the results of this study, detailed descriptions of the participants and the Swedish context is presented in the methodology section. This allows readers to assess the transferability of the results, as recommended by Kvale & Brinkmann [38] and Patton [54].

## Conclusion

In conclusion, local decision-makers have a favorable outlook on welfare technology, considering it a sustainable solution to the persistent challenges of funding and staff shortages in elder care. They recognize its potential to alleviate staff burdens by streamlining administrative tasks and enhancing the independence and engagement of older adults. Nonetheless, the implementation of welfare technology in elder care is not without bureaucratic hurdles that need to be addressed.

## Data Availability

Examples in the form of quotations from the generated and analyzed data during this study are included in this published article.
